# Bioprospecting Fungal Biocontrol Agents from Florida Agroecosystems Against Celery Early Blight Caused by *Cercospora apii*

**DOI:** 10.3390/plants15131941

**Published:** 2026-06-24

**Authors:** Larissa Carvalho Ferreira, Katia Viana Xavier

**Affiliations:** Department of Plant Pathology, Everglades Research and Education Center, University of Florida, IFAS, Belle Glade, FL 33430, USA

**Keywords:** Everglades Agricultural Area, celery, *Apium graveolens*, integrated pest management, *Mucor nidicola*, *Mucor irregularis*, biological control of plant diseases, microbe–microbe interactions, plant pathology, fungal plant pathogen

## Abstract

Fungi are a promising source of biological control agents for the management of phytopathogens such as *Cercospora apii*, the causal agent of celery early blight. Exploring native fungal isolates associated with agroecosystems near celery production is essential for identifying biocontrol candidates and supporting sustainable, integrated disease management strategies. In this study, fungal isolates were obtained from leaves and soil samples collected across agricultural and natural environments and their antagonistic potential against *C. apii* was evaluated using in vitro assays. A total of 48 fungal isolates were screened for growth inhibition, of which 12 reduced pathogen colony size by more than 50% in vitro, representing five morphological and taxonomic groups: *Aspergillus fumigatus*, *Fusarium* spp., *Mucor* spp., *Neopestalotiopsis* sp. and *Nigrospora* sp. Notably, isolates exhibiting the highest antagonistic activity over time were predominantly derived from leaf samples (*p* < 0.0001). Two isolates, *Mucor nidicola* KX3187 and *M. irregularis* KX3197, consistently showed strong inhibition of *C. apii* in vitro (up to 85%), and *M. nidicola* significantly suppressed disease development *in planta*. This preliminary study identifies *Mucor nidicola* KX3187 as a potential biocontrol candidate that showed promising activity in greenhouse trials for celery early blight and provides a foundation for future studies to further evaluate its potential as a component of sustainable disease management strategies.

## 1. Introduction

Celery early blight, also known as Cercospora leaf spot, is one of the most economically important foliar diseases of celery (*Apium graveolens* L. var. *dulce*), causing lesions that reduce photosynthetic area and render celery unmarketable [1]. In the United States, celery production is concentrated in California, Michigan, and Florida, with the latter serving as a major producer of celery fresh market during the winter and spring seasons. In this region, *Cercospora apii* Fresen., the causal agent of celery early blight, is one of the most destructive foliar pathogens affecting celery crops, resulting in economic losses up to 100% [1,2,3]. Although the disease is primarily attributed to *Cercospora apii* sensu stricto [1,4], studies have revealed the presence of novel species, such as *C. apiicola*, in celery-growing regions of Venezuela, Korea, and Greece [5,6]. The occurrence of multiple *Cercospora* species associated with celery highlights the need to consider species-level differences when developing and implementing integrated disease management strategies. Current management of celery early blight in Florida rely almost exclusively on fungicide applications, typically on a weekly basis [1,7,8]. Conventional growers can utilize fungicides from diverse modes of action, most commonly demethylation inhibitors (DMIs), succinate dehydrogenase inhibitors (SDHIs), quinone outside inhibitors (QoIs), and multi-site fungicides (FRAC groups 3, 7, 11, and M) [9,10]. In contrast, organic growers are limited in available modes of action for rotation and primarily rely on copper-based products [7]. The strong dependence on chemical control poses significant challenges for both conventional and organic growers due to rising concerns including fungicide resistance, environmental impact, regulatory restrictions such as maximum residue limits, and the limited number of effective active ingredients available for organic production.

In the context of sustainable agriculture, there is an urgent need to reduce reliance on chemical inputs while maintaining effective disease control. This need is particularly critical in the Everglades Agricultural Area (EAA), where organic histosols (muck soils) support one of the largest celery production regions in Florida [11]. Intensive use of fungicides in these systems raises concerns about environmental contamination, soil health degradation, and impacts on adjacent sensitive ecosystems of the greater Everglades [12,13,14]. Reducing chemical inputs while preserving crop productivity is therefore essential to protect these unique soils and surrounding natural resources. Biological control is a cornerstone of integrated pest management and is increasingly explored as a viable alternative [15,16]. A major strategy within biological control is the use of fungal biocontrol agents (BCAs), which are naturally occurring organisms often well-adapted to specific environments and capable of suppressing pathogens through multiple mechanisms of action [17,18]. These mechanisms include direct antagonism through antibiosis, mycoparasitism, and nutrient competition, as well as indirect mechanisms such as plant growth promotion and the induction of host defense responses [19,20]. Notable fungal genera with proven or potential biocontrol activity include *Trichoderma*, *Clonostachys*, *Penicillium, Aspergillus* and *Mucor* [21,22,23,24,25]. Some of these fungi produce volatile organic compounds (VOCs) or extracellular enzymes, such as chitinases and glucanases, that degrade pathogen cell walls or interfere with their development [26,27,28].

Despite these promising attributes, one of the key challenges in implementing fungal BCAs lies in the variability of their efficacy under field conditions [29,30]. Factors such as local microclimate, soil microbiome, and formulation stability may compromise their performance [31,32,33]. A plausible hypothesis is that locally adapted microbial strains may provide more consistent biocontrol performance due to their ecological fitness within specific agroecosystems. However, local adaptation was not experimentally tested in this study, and this concept requires direct comparative evaluation across environments and strains.

This study aims to fill this gap by identifying fungal BCAs isolated from celery production systems in south Florida and investigating their biocontrol potential under *in vitro* and greenhouse conditions. Because accurate pathogen identification is essential for meaningful biocontrol screening, particularly within a taxonomically complex genus such as *Cercospora*, this study also aims to confirm the identity of the *Cercospora* species present in the EAA. The specific objectives were (1) to isolate *Cercospora* sp. from symptomatic celery, confirm its pathogenicity through Koch’s postulates, and verify its identity using molecular phylogenetic analyses; (2) to identify potential fungal biocontrol agents from commercial celery systems and natural environments; (3) to assess their antagonistic activity against *Cercospora* sp. in vitro; (4) to identify representative isolates using molecular phylogenetic analysis and investigate their potential modes of action, including volatile-mediated growth inhibition; and (5) to evaluate early blight suppression under greenhouse conditions. Together, these findings contribute to the development of fungal-based biocontrol strategies that may support integrated management of celery early blight, pending future validation under field conditions.

## 2. Results

### 2.1. Koch’s Postulate and Molecular Identification of Cercospora apii

**Figure 1:**
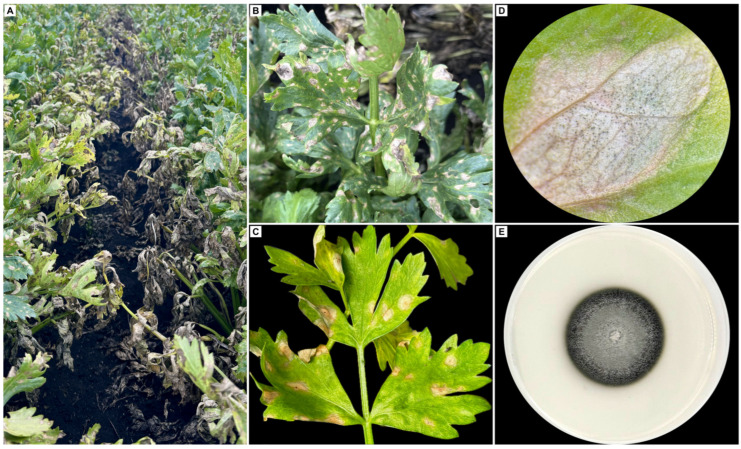
Characterization of *Cercospora apii* isolate KX493. (**A**) Early blight symptoms on celery plants 55 days after inoculation with isolate KX493 under field conditions, (**B**) and an individual plant showing mature lesions with extensive necrosis and sporulation. (**C**) Early blight symptoms on celery leaves 25 days after inoculation with isolate KX493 under greenhouse conditions, (**D**) and typical lesion morphology observed under a stereoscope at 35× magnification. Non-inoculated control plants were included in all experiments and remained symptom-free with healthy green foliage and no foliar lesions throughout the observation period. (**E**) A Petri dish showing 14-day-old PDA culture of the isolate.

The isolate KX493, obtained in this study, induced typical early blight symptoms on celery under both greenhouse and field conditions (Figure 1). Field inoculations on adult plants (eight weeks after transplanting) resulted in mature lesions with extensive necrosis and abundant sporulation when left untreated with fungicides, demonstrating the high virulence of this isolate (Figure 1A,B). Under greenhouse conditions, KX493 caused characteristic leaf symptoms (Figure 1C), and lesions observed under a stereoscope displayed typical lesion morphology (Figure 1D). The pathogen was successfully re-isolated from symptomatic tissue, and pure cultures exhibited typical colony morphology on PDA after 14 days (Figure 1E), thereby fulfilling Koch’s postulates.

**Figure 2:**
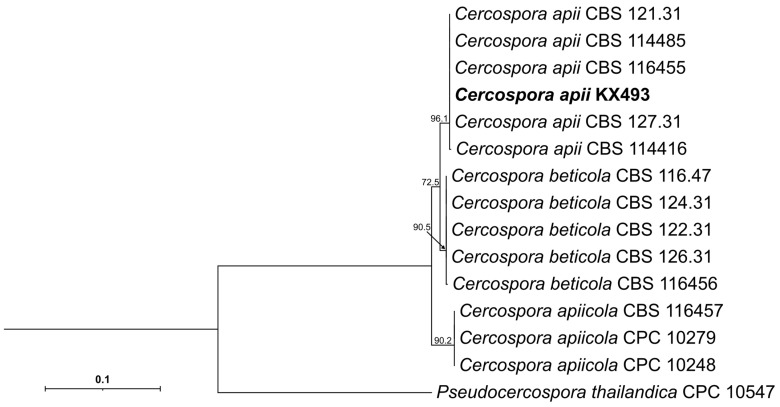
Maximum likelihood phylogenetic tree of *Cercospora apii* and related species showing taxonomic placement of isolate KX493 (bold) based on concatenated ITS, *act*, and *cmdA* sequence data. Bootstrap support values greater than 70% are shown above corresponding branches or indicated by arrow.

Molecular analysis using multilocus sequence data confirmed the identification of KX493 as *Cercospora apii*, with species-level discrimination primarily provided by the *act* gene (Figure 2). In the ITS region, KX493 showed 100% sequence identity with all *C. apii*, *C. beticola*, and *C. apiicola* isolates included in the phylogenetic tree (Figure 2). For the *cmdA* gene, KX493 shared 100% identity with *C. apii* and *C. beticola* isolates, and 94.74% identity with *C. apiicola* isolates. The *act* gene provided the greatest discriminatory power among the loci analyzed. KX493 exhibited 100% similarity with all *C. apii* isolates in the phylogenetic tree, except for strain CBS 114416 (99.64%). In contrast, similarity with *C. beticola* isolates was lower (95.71%, except CBS 116456 at 95.36%), and lowest with the *C. apiicola* isolates (93.21%). To our knowledge, this study represents the first multilocus molecular characterization of a *Cercospora apii* isolate obtained from celery in the Everglades Agricultural Area of Florida. Based on its confirmed identity as *C. apii* and pathology on celery, isolate KX493 was selected for subsequent experiments.

### 2.2. Diversity and Morphological Classification of Fungal Isolates

**Figure 3:**
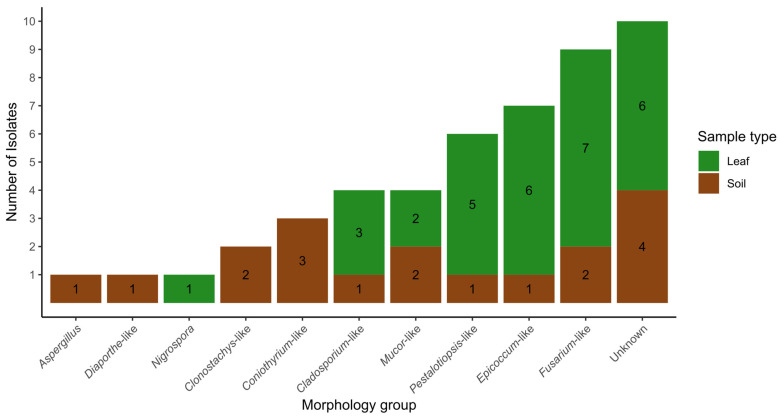
Morphology-based classification of isolates. Bars represent the number of isolates grouped by morphological similarity, separated by source (soil or leaf). Numbers displayed within the bars indicate the number of isolates from each source.

A total of 48 fungal isolates were obtained from five locations in south Florida (Table S1). The number of isolates collected was relatively consistent across sites, except for the conventional celery farm, from which only four isolates were obtained. Of the total, 30 isolates were recovered from leaf tissue and 18 from soil samples (Figure 3). Based on morphological classification using bright-field microscopy, the isolates were grouped into ten morphological categories plus one unidentified group. The five most represented categories were *Fusarium* (*n* = 9), *Epicoccum*-like (*n* = 7), *Pestalotiopsis*-like (*n* = 6), *Mucor*-like (*n* = 4), and *Cladosporium*-like (*n* = 4). These dominant groups were recovered from both soil and leaf sources. Less frequent taxa included *Coniothyrium*-like, *Clonostachys*-like, *Diaporthe*-like and *Aspergillus* groups, all recovered exclusively from soil, and *Nigrospora*, represented by a single leaf isolate.

### 2.3. Growth Inhibition Potential of BCAs in Dual Culture

The biocontrol potential of the fungal isolates (*n* = 48) was assessed through an in vitro antagonism assay against *Cercospora apii* isolate KX493. Fungal growth was monitored at 3, 7, 10, and 14 days post plating and compared to control plates containing *C. apii* alone (Figures S1–S4). On day 3, there were minimal differences in colony diameter between treatments and the control. By day 7, however, *C. apii* colonies in the control plates were noticeably larger than most treatment groups, and this difference became more pronounced by day 14, when the control plates exhibited the largest colony diameters.

To quantify these observed differences, inhibition of *C. apii* was analyzed statistically and was significantly influenced by time and by its interaction with sample type, while sample type alone was not significant (Type III Wald χ^2^ test; Time: χ^2^ = 400.95, *p* < 0.0001; Sample type: χ^2^ = 0.69, *p* = 0.2275; Sample type × Time: χ^2^ = 49.14, *p* < 0.0001). Inhibition increased over time for both groups, but the magnitude of inhibition differed between leaf- and soil-derived isolates depending on the time point (Table 1). At day 3, inhibition did not differ significantly between sample types (*p* = 0.4101). However, from day 7 onward, isolates obtained from leaf tissues exhibited significantly greater inhibition than those from soil, with differences becoming more pronounced over time (day 7: *p* = 0.0101; day 10: *p* = 0.0004; day 14: *p* < 0.0001). Estimated marginal means indicated that within our collection, inhibition by leaf-derived isolates was 23.36% greater than soil-derived isolates at day 14 (*p* < 0.0001).

**Figure 4:**
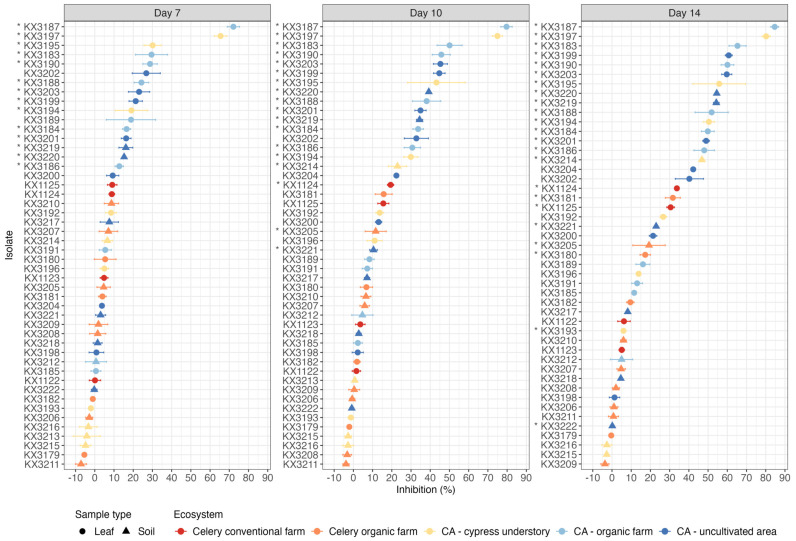
Percentage of *Cercospora apii* mycelial growth inhibition by fungal isolates in dual-culture assays, measured at 7, 10, and 14 days post plating. Isolates marked with an asterisk (*) made direct mycelial contact with *C. apii*. Point color indicates the ecosystem of origin, with “CA” referring to conservation areas. Bars represent standard errors of the mean inhibition values.

Physical contact between the BCA isolates and *C. apii* increased over time. By day 3, two isolates (KX3187 and KX3197) had reached the pathogen. This increased to 14 BCA isolates by day 7, 18 by day 10, and 23 by day 14 (Figure 4). In terms of growth inhibition, most isolates showed limited antagonism by day 3, with only 10 isolates exhibiting greater than 10% inhibition and a maximum of 36.6% inhibition observed (Figure S5). A clearer trend emerged between days 7 and 14; however, most isolates still showed inhibition below 20%. The top-ranked isolates across time points, those with the highest and most consistent effects, were predominantly isolated from leaves collected in the conservation areas (Figure 4). These top-ranked isolates also established contact with the *C. apii* colony early on, suggesting that rapid growth and competitive exclusion of space may play an important role in their antagonistic mechanism. Isolate KX3187 exhibited the highest level of inhibition, reaching up to 85% of growth inhibition by day 14.

**Figure 5:**
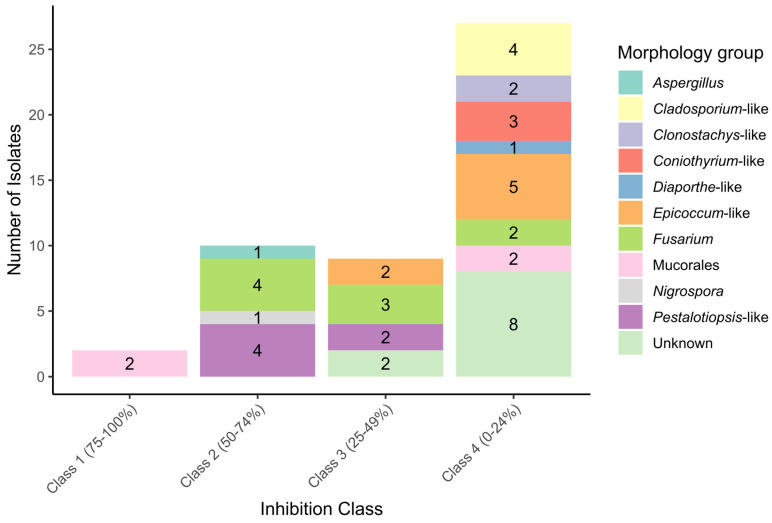
Inhibition classes by morphological group of fungal isolates at 14 days post plating against *Cercospora apii*. Each stacked bar represents the total number of isolates within an inhibition class, grouped by morphological classification. Inhibition was assessed using an in vitro dual-culture assay and expressed as percentage growth inhibition relative to the pathogen control. Inhibition classes were defined as follows: Class 1 (75–100% inhibition), Class 2 (50–74%), Class 3 (25–49%), and Class 4 (0–24%). Numbers within the bars indicate the count of isolates belonging to each morphological group.

**Figure 6:**
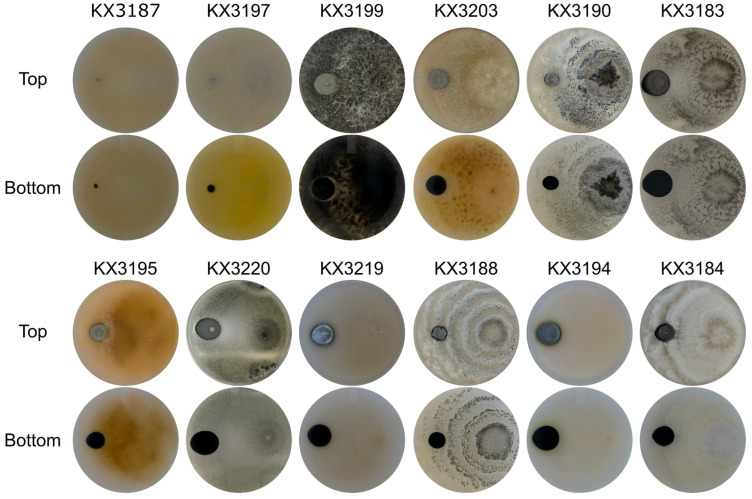
Representative dual-culture plates showing inhibition of *Cercospora apii* mycelial growth by fungal isolates, 14 days old. In each plate, *C. apii* was inoculated on the left side and the biocontrol agent candidate on the right. All isolates caused ≥50% inhibition compared to the control, which contains *C. apii* alone.

On day 14, fungal isolates displayed a broad range of growth inhibition activity against *C. apii*, which was categorized into four inhibition classes (Figure 5). Class 1, representing the highest level of antagonism (75–100% inhibition), included two isolates belonging to the *Mucor*-like group. Class 2 (50–74% inhibition) comprised 10 isolates spanning four morphological groups: *Fusarium*-like (*n* = 4) and *Pestalotiopsis*-like (*n* = 4) were the most frequent, followed by *Aspergillus* (*n* = 1) and *Nigrospora* (*n* = 1). Class 3 (25–49% inhibition) included nine isolates: *Epicoccum*-like (*n* = 2), *Fusarium*-like (*n* = 3), *Pestalotiopsis*-like (*n* = 2) and two morphologically unidentified isolates. The majority of isolates (*n* = 27), which caused the least inhibition (Class 4 [0–24% inhibition]), were distributed across eight morphological groups, with the most frequent being unidentified isolates (*n* = 8), followed by *Epicoccum*-like (*n* = 5), *Cladosporium*-like (*n* = 4), *Coniothyrium*-like (*n* = 3), *Clonostachys*-like (*n* = 2), *Fusarium*-like (*n* = 2), *Mucor*-like (*n* = 2), and *Diaporthe*-like (*n* = 1). These results indicate that strong antagonistic activity was limited to a few isolates, while moderate to low inhibitory potential was observed across a wide range of morphological groups. Isolates showing >50% growth inhibition (Classes 1 and 2) were considered promising candidates for further evaluation, as in vitro performance does not guarantee biocontrol efficacy *in planta*. All isolates in Classes 1 and 2 are shown in Figure 6.

### 2.4. Molecular Characterization of Selected Isolates and Volatile Organic Compounds (VOCs)

Isolates that demonstrated >50% growth inhibition in dual-culture assays (Figure 6), were selected for molecular identification. Phylogenetic analyses based on the internal transcribed spacer (ITS), large subunit (LSU), and elongation factor 1-α (TEF1) gene regions enabled taxonomic placement of 12 isolates (Figures S6–S10). Species-level identification was confirmed for six isolates: one isolate (KX3220) as *Aspergillus fumigatus*; three isolates (KX3195, KX3194, and KX3203) as *Fusarium mucidum*; one isolate (KX3197) as *Mucor irregularis*; and one isolate (KX3187) as *Mucor nidicola*.

The remaining isolates could not be resolved to the species level. Isolate KX3219 was identified as *Fusarium* sp., clustering closely with *F. oryzicola* and *F. arcuatisporum*. Four isolates (KX3183, KX3184, KX3188, and KX3190) were identified as *Neopestalotiopsis* spp. Additionally, isolate KX3199 was identified as *Nigrospora* sp., clustering closely with *N. hainanensis* and *N. manihoticola*.

**Figure 7:**
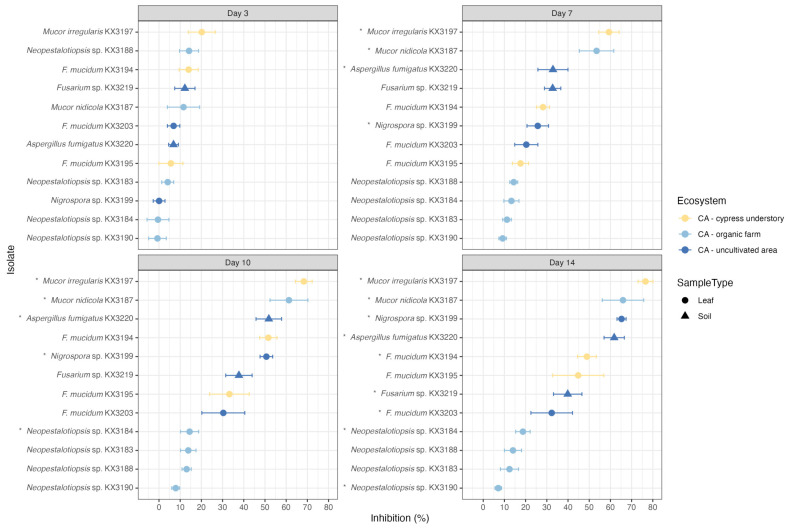
The percentage of *Cercospora apii* growth inhibition in the in vitro VOC dual-plate assay, measured at 3, 7, 10, and 14 days post plating. Isolates marked with an asterisk (*) indicate instances in which direct mycelial contact with *C. apii* occurred due to overgrowth across the plate interface. Point color indicates the ecosystem of origin, with “CA” referring to conservation areas. Bars represent standard errors of the mean inhibition values.

To evaluate the potential role of volatile organic compounds (VOCs) in fungal antagonism, the selected isolates were further tested using a sealed dual-plate assay. Inhibition of *C. apii* was highly variable among isolates (Figure 7). On day 3, inhibition was generally low across all isolates, with a maximum of 20.2%. By day 7, inhibition increased modestly, ranging from 9.2% to 32.8%. Only *F. mucidum* KX3194 exceeded 50% growth inhibition by day 10 (51.6%). By day 14, however, *F. mucidum* KX3194 had grown across the plate interface, and inhibition among isolates without mycelial contact ranged from 12.4% to 44.8%, with no isolate surpassing the 50% threshold through VOC-mediated effects alone.

However, it is important to note that five isolates that exhibited more than 50% of growth inhibition by day 14 (*M. nidicola* KX3187, *M. irregularis* KX3197, *Nigrospora* sp. KX3199 and *A. fumigatus* KX3220) grew across the plate interface and colonized the *C. apii* plate prior to the final time point. Consequently, inhibition observed for these isolates cannot be attributed solely to volatile-mediated effects, as direct mycelial interaction and overgrowth may also have contributed to pathogen suppression.

### 2.5. Growth Inhibition Efficacy and Colonization Potential of Top BCA Isolates in Planta

The two most promising fungal isolates identified from in vitro assays, *M. nidicola* KX3187 and *M. irregularis* KX3197, were selected for further evaluation *in planta*. To assess their pathogenicity on celery, spore suspensions were sprayed onto healthy plants, and plant responses were visually monitored over a 5-week period. No symptoms or anomalies were observed on the treatments or the mock-inoculated controls, indicating that neither isolate is pathogenic to celery.

To investigate colonization ability, re-isolations were performed two weeks post inoculation using four different methods targeting epiphytic and endophytic presence. No fungal colonies were recovered from any treatment except a single replicate of *M. irregularis* KX3197 obtained from surface-sterilized tissue (Figure S11). Because this recovery was not consistently reproduced across replicates, no conclusions regarding stable endophytic or epiphytic colonization could be drawn.

**Figure 8:**
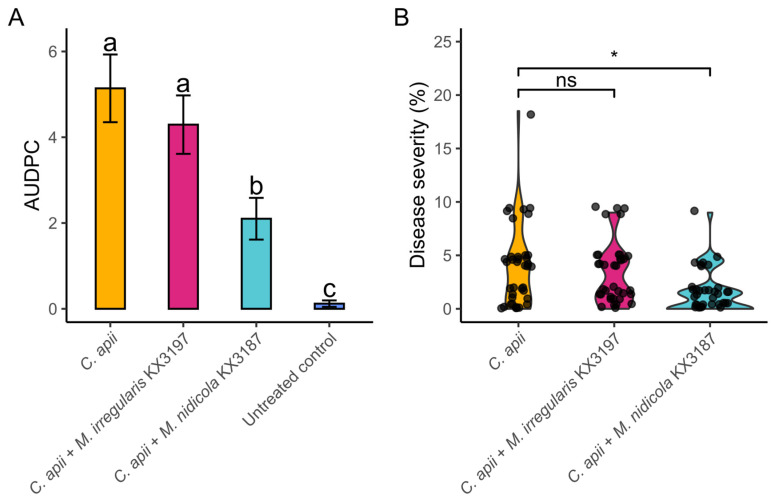
Disease suppression of *Cercospora apii* by fungal isolates *in planta*. (**A**) The Area Under the Disease Progress Curve (AUDPC) calculated from weekly disease assessments for three consecutive weeks after symptom onset (3–5 weeks post-inoculation) for celery plants inoculated with *C. apii* alone (inoculated untreated control), co-inoculated with *Mucor nidicola* KX3187 or *M. irregularis* KX3197 or maintained as a non-inoculated untreated control. Bars represent mean AUDPC values, and error bars indicate standard errors of the mean. Different letters above bars indicate significant differences among treatments based on a linear model followed by Fisher’s LSD test (*p* < 0.05). (**B**) Violin plots showing disease severity at 5 weeks post inoculation (final assessment). Individual points represent severity scores expressed as midpoints of the Horsfall–Barratt scale. Statistical analysis was performed using the cumulative link model (CLM) with pairwise comparisons via estimated marginal means. An asterisk above the bracket indicates a significant difference compared with the inoculated control (*p* = 0.001); “ns” indicates no significant difference (*p* > 0.05).

The biocontrol efficacy of KX3187 and KX3197 was further evaluated in a greenhouse trial in which celery plants were inoculated with *C. apii* followed by a single application of BCAs. Plants treated with *Mucor nidicola* KX3187 showed a statistically significantly lower Area Under The Disease Progress Curve (AUDPC) compared with the *C. apii*-only treatment (inoculated untreated control), while non-inoculated untreated control plants exhibited negligible disease levels (Figure 8A). *Mucor irregularis* KX3197 showed a numerical reduction in disease severity but this effect was not statistically significant. The effect of this single application was maintained through 5 weeks post inoculation, with plants treated with *M. nidicola* KX3187 still exhibiting significantly reduced disease severity in comparison to *C. apii*-only treatment (inoculated untreated control) (Figure 8B), demonstrating *in planta* antagonistic activity.

## 3. Discussion

Early blight, caused by *Cercospora apii*, is a significant foliar disease of celery in Florida, capable of reducing yield and compromising product quality. Accurate pathogen identification is therefore essential for meaningful disease management and biocontrol screening, especially within a taxonomically complex genus such as *Cercospora*. In this study, we confirmed the pathogenicity of isolate KX493 through Koch’s postulates and verified its identity using multilocus phylogenetic analyses, ensuring that subsequent biocontrol evaluations targeted the correct pathogen.

Given that the efficacy of fungal biological control agents (BCAs) can be highly context-dependent, locally adapted strains may offer more reliable disease suppression due to their ecological fitness within specific agroecosystems. Motivated by this hypothesis, we isolated 48 fungal BCAs from celery production systems and adjacent natural environments in south Florida and evaluated their antagonistic potential against *C. apii*. Within our collection, several isolates exhibiting comparatively high growth inhibition activity were obtained from leaf samples collected in conservation areas. However, because isolate recovery and sampling intensity differed among environments, these observations should be interpreted cautiously and do not establish that conservation-associated fungi are inherently more antagonistic than isolates obtained from other sources. Similar studies have reported that native isolates of fungi such as *Trichoderma* and *Aspergillus* spp. can exhibit strong biocontrol activity compared to non-native or commercial strains [34,35]. Collectively, these findings suggest that plants in conservation areas may represent useful sources of fungal diversity for future bioprospecting efforts, although broader and more balanced sampling will be needed to robustly compare environments as sources of BCAs.

To assess the biocontrol potential of these isolates, we conducted in vitro dual-culture assays to evaluate their antagonistic activity against *C. apii*. By day 14, a total of 23 isolates had made physical contact with the pathogen, many of which significantly inhibited its growth. Among them, two *Mucor* spp. isolates (KX3187 and KX3197) stood out for their early and consistent suppression, reaching inhibition rates of up to 85%. This is consistent with growing evidence that species within the genus *Mucor*, including *M. hiemalis* and *M. moelleri*, have shown effective biocontrol mechanisms including pathogen suppression and plant growth promotion [23,36]. Additionally, *Mucor indicus* has been recognized for its potential in industrial biotechnology, underscoring the broader applicability of this genus in agriculture and related fields [37]. While these assays provide a valuable first step for identifying potential antagonists under controlled conditions, they represent an artificial system that may not fully capture the ecological complexity of plant–microbe–pathogen interactions in natural environments.

To investigate and characterize the most promising isolates, we performed molecular identification using ITS and LSU sequences, and TEF1 when available. The analysis confirmed some isolates at the species level (e.g., *Mucor nidicola*, *M. irregularis*, *Aspergillus fumigatus*, and *Fusarium mucidum*), whereas others were resolved only to the genus level (*Fusarium*, *Nigrospora*, and *Neopestalotiopsis* spp.), reflecting limitations in taxonomic resolution for certain groups based on the markers used. However, not all identified species are suitable for biocontrol applications due to safety concerns. *Aspergillus fumigatus* is a known opportunistic human pathogen and respiratory allergen [38,39], making it unsuitable for agricultural applications. Similarly, *Fusarium* species may pose risks through potential phytopathogenicity toward non-target crops or mycotoxin production [40,41]. Therefore, our biocontrol evaluation focused primarily on *Mucor* species (*M. nidicola* and *M. irregularis*), which showed strong antagonistic activity and have a more favorable safety profile. Future biocontrol development should prioritize thorough biosafety assessments including evaluation of potential pathogenicity, allergenicity, and toxin production before considering any isolate for field applications. Members of suitable genera have been widely reported in the literature to include strains with antagonistic activity and to produce diverse secondary metabolites with antimicrobial properties. For example, *Mucor moelleri* have been reported to inhibit plant pathogens such as *Athelia rolfsii* and *Colletotrichum gloeosporioides* through the production of VOCs and lytic enzymes [23]. Similarly, *Neopestalotiopsis* spp. have been associated with the production of antimicrobial metabolites such as eugenol and other compounds with inhibitory activity against a range of microorganisms, such as *Candida albicans*, *Aspergillus niger*, *Bacillus cereus*, and *Staphylococcus epidermidis* [42]. In addition, atoxigenic *Aspergillus* strains have also been reported to suppress plant pathogens (e.g., *Botrytis cinerea*, *Fusarium* spp., *Macrophomina phaseolina*, *Rhizoctonia solani*, *Sclerotinia sclerotiorum*) through secondary metabolites, enzyme secretion, and competition [43]. However, it is important to emphasize that these functional traits are genus-level generalizations derived from previous studies and do not necessarily reflect the specific mechanisms of the isolates characterized in this work. The metabolic and enzymatic traits responsible for the antagonistic activity observed in this study remain to be determined experimentally.

To further characterize antagonistic activity, selected isolates were evaluated in a sealed dual-plate assay intended to assess the potential contribution of volatile metabolites to pathogen suppression. Several isolates substantially reduced *C. apii* growth in this system. However, interpretation of these results is limited because multiple isolates produced aerial mycelium that crossed the plate interface and established growth on the pathogen-containing plate during the assay. Therefore, the observed inhibition cannot be conclusively attributed to VOC production alone, as physical colonization and direct fungal interactions likely also contributed to pathogen suppression. Accordingly, these findings should be considered preliminary evidence of antagonistic activity under sealed conditions rather than definitive proof of VOC-mediated inhibition. Future studies employing improved physical separation systems or analytical characterization of emitted volatiles will be necessary to clarify the role of VOCs in the antagonistic activity of these isolates.

Building on these insights, we evaluated the biocontrol potential of select isolates *in planta*. The greenhouse trials confirmed that *Mucor nidicola* KX3187 significantly reduced disease development. *Mucor irregularis* KX3197 did not demonstrate statistically significant biocontrol activity. Neither isolate caused disease symptoms in celery. Re-isolation attempts resulted in only a single recovery of *M. irregularis* KX3197 from surface-sterilized tissue, and this observation was not reproducible across replicates. Therefore, the present study did not provide sufficient evidence for consistent detectable endophytic or epiphytic colonization under the experimental conditions tested. While the culture-based recovery methods used in this study are suitable for detecting actively growing fungi, they may underestimate low-abundance colonization. Consequently, although our results suggest limited persistence or establishment of these isolates within plant tissues, low-level colonization below the detection threshold of the assay cannot be excluded. Future studies using species-specific molecular approaches could provide greater sensitivity for confirming colonization patterns.

Despite promising findings, several limitations warrant discussion. First, overgrowth in the VOC assay hindered interpretation, highlighting the need for more refined experimental setups to separate physical from chemical antagonism in vitro. Second, the antagonistic activity observed in vitro may not fully translate to *in planta* conditions, where environmental variability and microbial complexity can influence BCA performance. Finally, re-isolation attempts yielded minimal and inconsistent recovery of inoculated isolates, limiting conclusions regarding colonization dynamics and long-term persistence.

Future studies should prioritize evaluating *M. nidicola* KX3187 and other promising isolates under expanded greenhouse and field conditions. Isolates that consistently suppress disease across environments could then be further characterized using genomic or metabolomic approaches to investigate the mechanisms associated with antagonistic activity. Additional studies evaluating formulation strategies that improve survival and activity under field conditions may also be warranted. Field trials will ultimately be necessary to determine the consistency, persistence, and practical applicability of these isolates within pest management programs, particularly for organic production systems where biological options are limited. In addition, investigating interactions between these BCAs and the resident plant-associated microbiome may help explain variability in performance and identify ecological factors that influence their reliability and long-term success in integrated disease management systems.

Overall, this preliminary study demonstrates the feasibility of identifying fungal BCA candidates for celery early blight, confirming both pathogen identity and BCA antagonistic potential through multilocus molecular analyses, in vitro assays, and greenhouse trials. However, the mechanism of action responsible for disease suppression by *M. nidicola* KX3187 remains unknown. Our VOC assays could not definitively separate volatile-mediated effects from physical interactions, and colonization studies provided insufficient evidence for stable plant-associated persistence. Without confirmed colonization capacity or understood mechanisms of action, the long-term field efficacy and practical applicability of *M. nidicola* KX3187 remain uncertain. The findings suggest potential for developing fungal-based biocontrol strategies, but substantial additional research on mechanism of action, plant colonization dynamics, and field performance will be required before practical implementation can be considered.

## 4. Materials and Methods

### 4.1. Cercospora sp. Isolation and Koch’s Postulate

Symptomatic celery leaves with typical early blight symptoms were collected from research field plots at the University of Florida Everglades Research and Education Center (EREC, Belle Glade, FL, USA). The transition tissue between lesion and green tissue was cut, surface-sterilized (70% ethanol for 30 s, 1.2% bleach for 3 min, followed by three rinses in autoclaved deionized water) and plated on potato dextrose agar (PDA; 39 g L^−1^; BD Difco™, Sparks, MD, USA). The resulting isolate was designated as KX493 and preserved at −80 °C in 15% (*v*/*v*) glycerol.

For inoculum preparation, KX493 was cultured on V8-juice agar using the seeding method [44]. Ten-day-old cultures were flooded with sterile distilled water, and spores were gently dislodged using a sterile pestle. The suspension was homogenized for 1 min, adjusted to 2.5 × 10^5^ CFU (colony-forming units representing viable propagules including spores and mycelial fragments) mL^−1^, and supplemented with 0.01% (*v*/*v*) Tween 20. Celery seedlings cultivar Duda 30 (susceptible to early blight), grown as tray transplants for approximately 12 weeks at the nursery, were then established in pots under greenhouse conditions. Three weeks after transplanting, plants were inoculated by spraying the spore suspension until runoff. Plants were subsequently monitored for symptom development, and typical early blight symptoms were observed. Symptomatic leaf tissue was then re-isolated, and the recovered fungus was examined using light microscopy to confirm morphological characteristics consistent with the original pathogen, thereby fulfilling Koch’s postulates. Similarly, to evaluate pathogenicity under field conditions, plants of the cultivar Duda 30 growing at EREC were inoculated eight weeks after transplanting using an inoculum with ~3.7 × 10^4^ CFU mL^−1^, prepared as described above. Following field inoculation, plants were monitored for symptom development.

### 4.2. Field Sampling and Isolate Collection of Biological Control Agents

**Figure 9:**
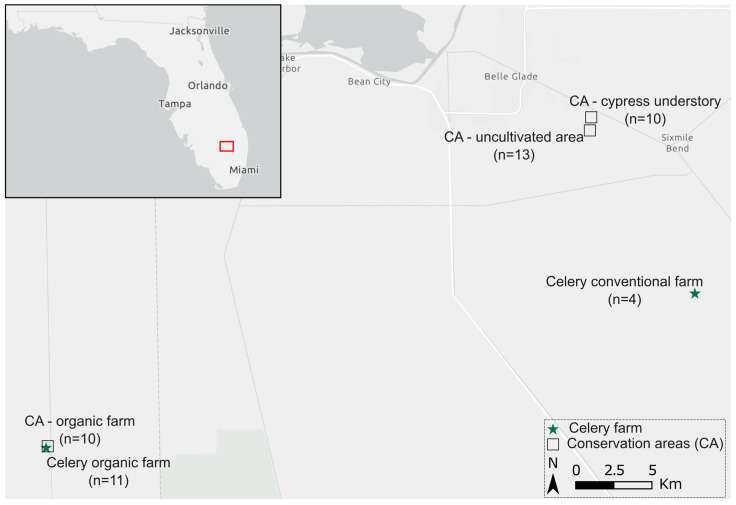
An overview of fungal biocontrol agent collection. Geographic metadata of sampling locations in Florida, with an inset map showing the focal region (red rectangle). Celery farms are indicated by green stars, and conservation areas (CAs) by hollow squares. Numbers in parentheses next to each location indicate the number of fungal strains isolated.

Sampling was conducted across five distinct locations in south Florida (Figure 9), including one organic and one conventional celery farm. From these commercial farms, samples were collected from asymptomatic plants located in areas with a known history of disease. A conservation area within the organic farm was also included, where perennial understory plants were sampled (Figure 9). Additionally, two sites at the University research station, EREC, were sampled: a cypress stand and an adjacent uncultivated area, both containing perennial understory vegetation (Figure 9). The uncultivated area, also known as “virgin land,” has not been agronomically cultivated for over four decades. At each location, sixteen plants were selected following a zig-zag sampling pattern. One fully developed (but non-senescent) leaf was collected per plant for fungal isolation. Leaves were transported to the laboratory and stored at 4 °C until processing. In parallel, approximately 20 g of soil was collected from the base or root zone of each sampled plant.

### 4.3. Fungal Isolation Procedures

Leaf samples were first washed in soapy water to remove debris, then thoroughly rinsed under running tap water. Using a sterilized scalpel, a small segment (~1 cm^2^) was excised from each representative leaf (one per plant) and placed adaxial side down onto quarter-strength PDA (QPDA; 9.75 g L^−1^) plates. For soil samples, approximately 5 g from each plant were pooled into four groups (plants 1–4, 5–8, 9–12, and 13–16) in separate Erlenmeyer flasks. Each flask received 50 mL of autoclaved deionized water and was agitated at 150 rpm for 30 min. Then, a 100 µL aliquot from each flask was subjected to serial dilution. The 10^−4^ dilution was plated on QPDA. All plates were placed on the benchtop at room temperature (23 ± 3 °C) under a 12 h light/12 h dark photoperiod. Colonies exhibiting distinctive morphology or larger growth were subcultured onto fresh QPDA and preserved at −80 °C in 15% (*v*/*v*) glycerol for long-term storage.

### 4.4. In Vitro Antagonism Assay Against Cercospora apii

To evaluate the antagonistic potential of fungal isolates against *Cercospora apii*, an in vitro dual-culture assay was performed. The *C. apii* strain KX493 was point-placed on one side of a Petri dish containing PDA using a sterile toothpick, while the candidate biocontrol isolate was point-placed on the opposite side. A standardized template was used to ensure consistent spacing and positioning of fungal isolates and *C. apii* placement points across all plates. Control plates contained *C. apii* only. Each fungal isolate was tested in five replicates (*n* = 5), and the experiment was repeated twice. Plates were sealed with parafilm, incubated in the dark at 23 ± 3 °C, and colony diameters were measured at 3, 7, 10, and 14 days post placement. For each plate, two perpendicular colony diameters were averaged to calculate mean growth. The percentage of growth inhibition was determined using the following formula: Inhibition (%) = (D_C_ − D_BCA_)/D_C_ × 100, where D_C_ is the mean diameter of *C. apii* in the control, and D_BCA_ is the mean diameter of *C. apii* in the presence of the biocontrol agent candidate. Based on inhibition values, isolates were categorized into four classes: Class 1 (75–100% inhibition), Class 2 (50–74%), Class 3 (25–49%), and Class 4 (0–24%). During evaluation, physical interactions between the fungal colonies were also recorded. Isolates were considered to have positive contact when colony convergence with *C. apii* occurred in two or more replicates. Contact status was recorded to aid in interpreting inhibition mechanisms, distinguishing between isolates that achieved high inhibition through spatial competition/physical interaction versus those that inhibited pathogen growth without direct contact.

Statistical analyses were conducted in R using linear mixed-effects models fitted with the lme4 package [45]. Inhibition (%) was modeled as a function of sample type, days post plating, and their interaction as fixed effects, with isolate and experiment included as random effects. Model assumptions and fit were evaluated using simulated residual diagnostics implemented in the DHARMa package [46]. The significance of fixed effects was assessed using Type III Wald χ^2^ tests with the Anova function from the car package [47]. Pairwise comparisons between sample types at each time point were performed using estimated marginal means with the emmeans package [48].

### 4.5. Morphological and Molecular Identification of BCAs

All fungal isolates were cultured on potato dextrose agar (PDA) and incubated at room temperature (23 ± 3 °C) for 20 days. Colony morphology was evaluated based on macroscopic traits including color, texture, margin, and sporulation pattern. Microscopic characteristics such as spore type, conidiophore morphology, and hyphal structure were examined using glass slide mounts under a Zeiss Primostar 3 microscope (Carl Zeiss Microscopy, Oberkochen, Germany). Isolates sharing similar colony and microscopic features were assigned to provisional morphological groups [49]. When precise genus-level identification was uncertain based on morphology alone, isolates were assigned to a provisional “-like” category (e.g., *Epicoccum*-like, *Cladosporium*-like, *Coniothyrium*-like) to indicate resemblance to a known genus without implying confirmed identity.

For molecular identification, isolates demonstrating >50% growth inhibition against *C. apii* in dual-culture assays (Classes 1 and 2) were subcultured from single spores or hyphal tips (in cases where sporulation was absent) to ensure purity. These pure cultures were preserved at −80 °C in a 15% (*v*/*v*) glycerol solution. Genomic DNA was extracted from actively growing mycelia using the SYNERGY 2.0 DNA Extraction Kit (OPS Diagnostics, Lebanon, NJ, USA), following the manufacturer’s protocol. Purified DNA was eluted using Monarch DNA elution buffer (New England Biolabs, Ipswich, MA, USA) and stored at −20 °C until further use.

The internal transcribed spacer (ITS) region was first amplified using primers ITS1F (CTTGGTCATTTAGAGGAAGTAA) [50] and ITS4 (TCCTCCGCTTATTATATGC) [51]. PCR reactions were performed in a Biometra thermal cycler (Analytik Jena, Jena-Göschwitz, Germany) under the following conditions: initial denaturation at 95 °C for 5 min, followed by 35 cycles of denaturation at 94 °C for 30 s, annealing at 51 °C for 30 s, and extension at 72 °C for 40 s. The program concluded with a final extension at 72 °C for 8 min, followed by 8 min at 15 °C. Amplified PCR products were purified and sequenced by MCLAB Molecular Cloning Laboratories (South San Francisco, CA, USA). Resulting sequences were manually curated and assembled in Geneious Prime (Version 2025.2.2). Consensus sequences were queried against the NCBI nucleotide (nr/nt) database using BLASTn (https://blast.ncbi.nlm.nih.gov) for preliminary genus-level identification. A second round of PCRs was performed to improve species-level resolution, following the same cycling conditions as for ITS but with annealing temperatures optimized for each target. For isolates belonging to *Aspergillus*, *Fusarium*, *Mucor*, *Neopestalotiopsis*, and *Nigrospora*, the large subunit (LSU) rRNA region was amplified using primers LR0R (TCCTCCGCTTATTGATATGC) and LR5 (ACCCGCTGAACTTAAG) [52], with an annealing temperature of 48 °C. For *Fusarium* isolates, the translation elongation factor 1-α (TEF1) gene was additionally amplified using primers EF1 (ATGGGTAAGGARGACAAGAC) and EF2 (GGARGTACCAGTSATCATGTT) [53], with an annealing temperature of 53 °C. For *Cercospora apii*, fragments of the calmodulin (*cmdA*) and actin (*act*) genes were amplified using primer pairs CAL-228F (GAGTTCAAGGAGGCCTTCTCCC)/CAL-737R (CATCTTTCTGGCCATCATGG), and ACT-512 (ATGTGCAAGGCCGGTTTCGC)/ACT-783R (TACGAGTCCTTCTGGCCCAT) with annealing temperatures of 56 °C and 59 °C, respectively.

Sequences were processed as previously described and were deposited in GenBank under the following accession numbers: PX655608-PX655616 for ITS, PX652380-PX652391for LSU, PX663052-PX663054 for TEF1, PX663051 for *cmdA*, and PX663050 for *act* (Table S1).

For phylogenetic analysis, reference sequences were retrieved from MycoBank, FUSARIOID-ID and reference studies [6,54,55,56,57,58,59,60,61,62]. Sequences were aligned using MAFFT v7.490 [63,64] in Geneious Prime, concatenated with Mesquite 4.01 [65], and analyzed with W-IQ-TREE [66] using 1000 bootstrap replicates. The best-fit substitution models for each partition were determined by ModelFinder [67] using the Bayesian Information Criterion (BIC). For each genus, the following sequence lengths and models were used: *Aspergillus* (528 bp ITS, 906 bp LSU; TIM2+F+G4, TIM+F+I+G4), *Cercospora* (483 bp ITS, 287 bp *cmdA*, 191 bp *act*), *Fusarium* (672 bp TEF1, 816 bp LSU; TNe+I+G4, K2P+I), *Mucor* (900 bp ITS, 930 bp LSU; TPM3+F+G4, TIM3+F+I+G4), *Neopestalotiopsis* (571 bp ITS, 819 bp LSU; TIM3e+G4, K2P), and *Nigrospora* (579 bp ITS, 858 bp LSU; TNe+G4, K2P). Maximum likelihood trees were generated under the selected models following initial parsimony tree reconstruction using the Phylogenetic Likelihood Library (PLL), and resulting phylogenies were visualized with iTOL [68].

### 4.6. Analysis of Volatile Organic Compounds (VOCs) In Vitro

The effect of VOCs produced by fungal BCAs on *Cercospora apii* was evaluated using dual-plate assays as described below. *Cercospora apii* strain KX493 was point-placed in the center of a PDA plate, while the BCA was point-placed at the center of a separate PDA plate. The two plates were stacked agar side to agar side and sealed together with parafilm to allow for only VOC exchange between them. Colony growth of *C. apii* was measured at 3, 7, 10, and 14 days post plating, as previously described, and the percentage of growth reduction was calculated using the same inhibition formula. The experiment was conducted twice.

### 4.7. Pathogenicity and Colonization Assay of Fungal Biocontrol Candidates

The pathogenicity of two fungal isolates, *Mucor nidicola* KX3187 and *Mucor irregularis* KX3197, was evaluated on celery plants via foliar inoculation. Plants were prepared as previously described. Inoculations were performed three weeks after transplanting. The inoculum was prepared by flooding one-week-old cultures with sterile water and gently dislodged mycelia with a mini-pestle. The resulting suspension was homogenized in a blender for 1 min. The final concentration was adjusted to 2.5 × 10^5^ CFU mL^−1^ and Tween 20 was added at 0.01% (*v*/*v*). Plants were sprayed until runoff with the inoculum suspension and immediately enclosed in plastic bags to maintain high humidity (RH > 90%) for 48 h. Control (mock-inoculated) plants were sprayed with a 0.01% Tween 20 solution and subjected to identical conditions. All plants were maintained under greenhouse conditions and monitored for five weeks post inoculation. The experiment followed a completely randomized block design with five plants per treatment and four blocks (*n* = 20 plants per treatment) and was conducted twice. Plants were observed daily for symptom development or visual deviations relative to mock-inoculated controls.

To determine whether isolates exhibited epiphytic or endophytic colonization, leaf samples were collected two weeks post inoculation. The oldest leaf from one plant per block per treatment (including controls) was excised and pooled by treatment. To assess epiphytic colonization, leaves were shaken in 250 mL of sterile distilled water for 2 min, and the adaxial surface was gently pressed onto PDA for 30 s before removal. A 100 µL aliquot of the leaf wash was plated on PDA in four replicates. For endophytic colonization, one ~1 cm^2^ segment from each leaflet was excised and surface-sterilized as previously described and then plated on PDA. Stems from the same leaves were sterilized using the same procedure, cut longitudinally, and the internal surface was punctured with a sterile toothpick and point-inoculated onto PDA (four stem sections and four inoculation points per treatment). Plates were placed at room temperature (23 ± 3 °C) on the benchtop, and fungal growth was monitored daily based on morphological characteristics typical of *Mucor* spp.

### 4.8. Evaluation of Biocontrol Activity Against Cercospora apii Under Greenhouse Conditions

Inocula of *C. apii* KX493, *Mucor nidicola* KX3187 and *M. irregularis* KX3197 were prepared as previously described. One hour after *C. apii* inoculation, fungal BCAs (*Mucor* spp. KX3187 and KX3197) were applied using the same spraying procedure. Inoculated untreated control plants were inoculated with *C. apii* only (no BCA treatment), while non-inoculated untreated control plants were sprayed with 0.01% Tween 20 solution alone. The experiment followed a completely randomized block design, with four plants per treatment and five blocks (*n* = 20 plants per treatment), and was conducted twice under similar greenhouse conditions. Data from both experimental runs were pooled and analyzed together while accounting for experiment effects in the statistical models. Disease severity was assessed weekly for three consecutive weeks after symptom onset using the Horsfall–Barratt (HB) rating scale. The midpoints of each HB category were converted into percentages of affected tissue and used to calculate the Area Under the Disease Progress Curve (AUDPC) using the audpc function from the agricolae R package [69]. AUDPC data were analyzed using a linear model including treatment, experiment, and their interaction as fixed effects. Differences among means were evaluated using Fisher’s LSD test. Model assumptions were evaluated through residual diagnostics, including Q-Q plots, Shapiro–Wilk tests, and simulated residual analyses. Disease severity categories from the final assessment (5 weeks post inoculation) were analyzed using a cumulative link model (CLM) implemented in the ordinal R package. Treatment and experiment were included as fixed effects. Model fit and assumptions were evaluated using nominal and scale tests as well as diagnostic plots generated with the performance R package. Pairwise comparisons were performed using estimated marginal means obtained with the emmeans R package, and contrasts were tested against the inoculated control using the contrast function.

## 5. Conclusions

Overall, this study demonstrates the feasibility of identifying fungal isolates with antagonistic activity against celery early blight through multilocus molecular analyses, in vitro assays, and greenhouse evaluations. Specifically, *M. nidicola* KX3187 showed significant disease suppression in greenhouse trials. Further field studies will be needed to evaluate persistence, colonization dynamics, and efficacy across production environments.

## Figures and Tables

**Table 1:** Estimated marginal mean differences in inhibition (%) of *Cercospora apii* between leaf- and soil-derived isolates across time points (days post plating).

Days	Adjusted Mean Difference (%)	Standard Error	*p*
3	3.73	4.49	0.4101
7	12.41	4.68	0.0101
10	17.84	4.76	0.0004
14	23.36	4.88	<0.0001

## Data Availability

The original contributions presented in this study are included in the article/Supplementary Material. Further inquiries can be directed to the corresponding author.

## Supplementary Materials

The following supporting information can be downloaded at https://www.mdpi.com/article/10.3390/plants15131941/s1: Figure S1: Radial growth (mm) of *Cercospora apii* colonies in dual-culture assays with fungal isolates, measured at 3 days post inoculation. Isolates marked with an asterisk (*) made direct mycelial contact with *C. apii*. Boxplot colors indicate the ecosystem of origin, and boxes represent the median, interquartile range, and overall distribution of observed values; Figure S2. Radial growth (mm) of *Cercospora* apii colonies in dual-culture assays with fungal isolates, measured at 7 days post inoculation. Isolates marked with an asterisk (*) made direct mycelial contact with *C. apii*. Boxplot colors indicate the ecosystem of origin, and boxes represent the median, interquartile range, and overall distribution of observed values; Figure S3. Radial growth (mm) of *Cercospora apii* colonies in dual-culture assays with fungal isolates, measured at 10 days post inoculation. Isolates marked with an asterisk (*) made direct mycelial contact with *C. apii*. Boxplot colors indicate the ecosystem of origin, and boxes represent the median, interquartile range, and overall distribution of observed values; Figure S4. Radial growth (mm) of *Cercospora apii* colonies in dual-culture assays with fungal isolates, measured at 14 days post inoculation. Isolates marked with an asterisk (*) made direct mycelial contact with *C. apii*. Boxplot colors indicate the ecosystem of origin, and boxes represent the median, interquartile range, and overall distribution of observed values; Figure S5. Percentage of *Cercospora apii* mycelial growth inhibition by fungal isolates in dual-culture assays, measured at 3 days post inoculation. Isolates marked with an asterisk (*) made direct mycelial contact with *C. apii*. Point color indicates the ecosystem of origin, with “CA” referring to conservation areas. Bars represent standard errors of the mean inhibition values. Figure S6. The maximum likelihood phylogenetic tree based on concatenated ITS and LSU sequences, depicting the taxonomic placement of isolate KX3220 as *Aspergillus fumigatus* (highlighted in bold). Bootstrap support values > 70% are shown above the branches. *Penicillium digitatum* CBS 112082 was used as the outgroup; Figure S7. The maximum likelihood phylogenetic tree based on concatenated TEF1 and LSU sequences, depicting the taxonomic placement of *Fusarium* isolates (indicated in bold). Bootstrap support values > 70% are shown above the branches. *Fusarium graminearum* CBS 136009 was used as the outgroup; Figure S8. The maximum likelihood phylogenetic tree based on concatenated ITS and LSU sequences, depicting the taxonomic placement of *Mucor* isolates (indicated in bold). Bootstrap support values > 70% are shown above the branches. *Rhizopus homothallicus* CBS 336.62 was used as the outgroup; Figure S9. The maximum likelihood phylogenetic tree based on concatenated ITS and LSU sequences, showing the taxonomic placement of *Neopestalotiopsis* isolates (indicated in bold). Bootstrap support values > 70% are shown above the branches. *Discosia pseudoartocreas* CBS 136438 was used as the outgroup; Figure S10. The maximum likelihood phylogenetic tree based on concatenated ITS and LSU sequences, showing the taxonomic placement of *Nigrospora* isolates (indicated in bold). Bootstrap support values > 70% are shown above the branches. *Apiospora pseudoparenchymatica* LC7234 was used as the outgroup. Figure S11. Endophytic colonization assay of celery plants. (A) Representative celery leaf showing the sampling procedure: a ~1 cm^2^ segment was excised from each leaflet and surface sterilized prior to plating for endophytic isolation. (B) Representative PDA plates showing no fungal growth in the control (left) and emergence of a characteristic Mucor colony from one leaf segment (right). Table S1: Fungal isolates recovered from celery-associated ecosystems and used in this study. Information includes strain designation, sample type, ecosystem of origin, colony morphology, taxonomic identification, and GenBank accession numbers for the internal transcribed spacer (ITS), large subunit ribosomal RNA (LSU), translation elongation factor 1-α (TEF1), calmodulin (*cmdA*), and actin (*act*) gene regions.
