# Supplementary material for: Bioprospecting Fungal Biocontrol Agents from Florida Agroecosystems Against Celery Early Blight Caused by Cercospora apii
Source: Plants (Basel). 2026 Jun 24;15(13):1941. doi: 10.3390/plants15131941 (PMC13364228; doi:10.3390/plants15131941)
Supplement: Supplementary file 1 [file plants-15-01941-s001.zip › Supplementary Figures.pdf]

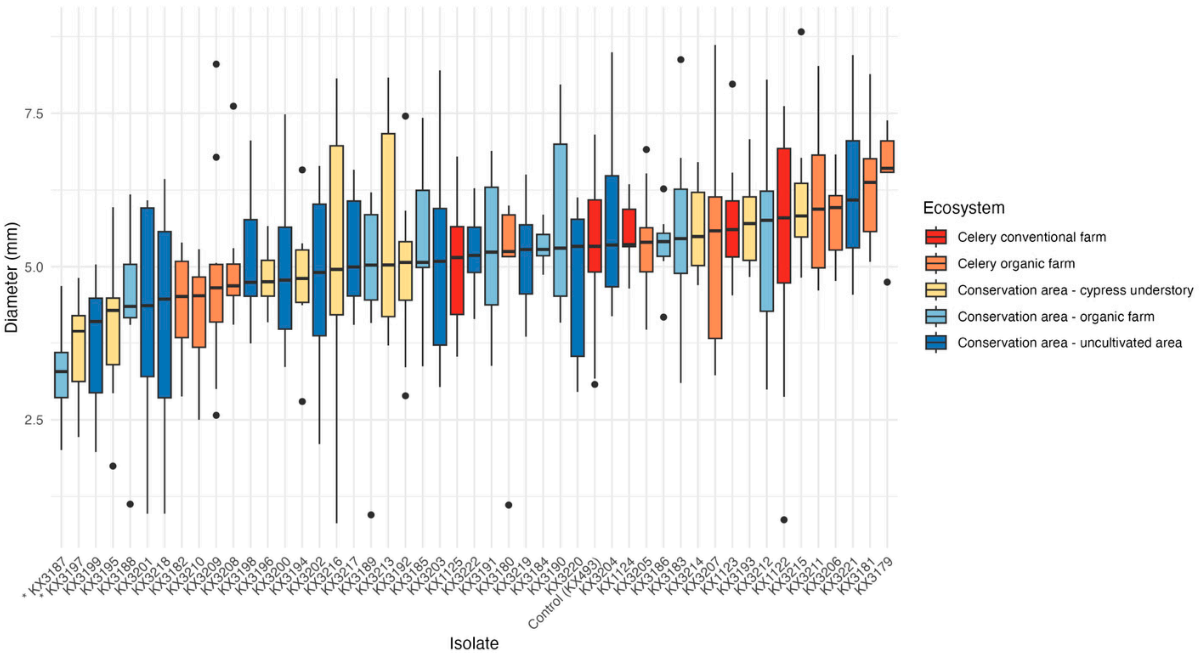

Figure S1. Radial growth (mm) of *Cercospora apii* colonies in dual-culture assays with fungal isolates, measured at 3 days post-inoculation. Isolates marked with an asterisk (*) made direct mycelial contact with *C. apii*. Boxplot colors indicate the ecosystem of origin, and boxes represent the median, interquartile range, and overall distribution of observed values.

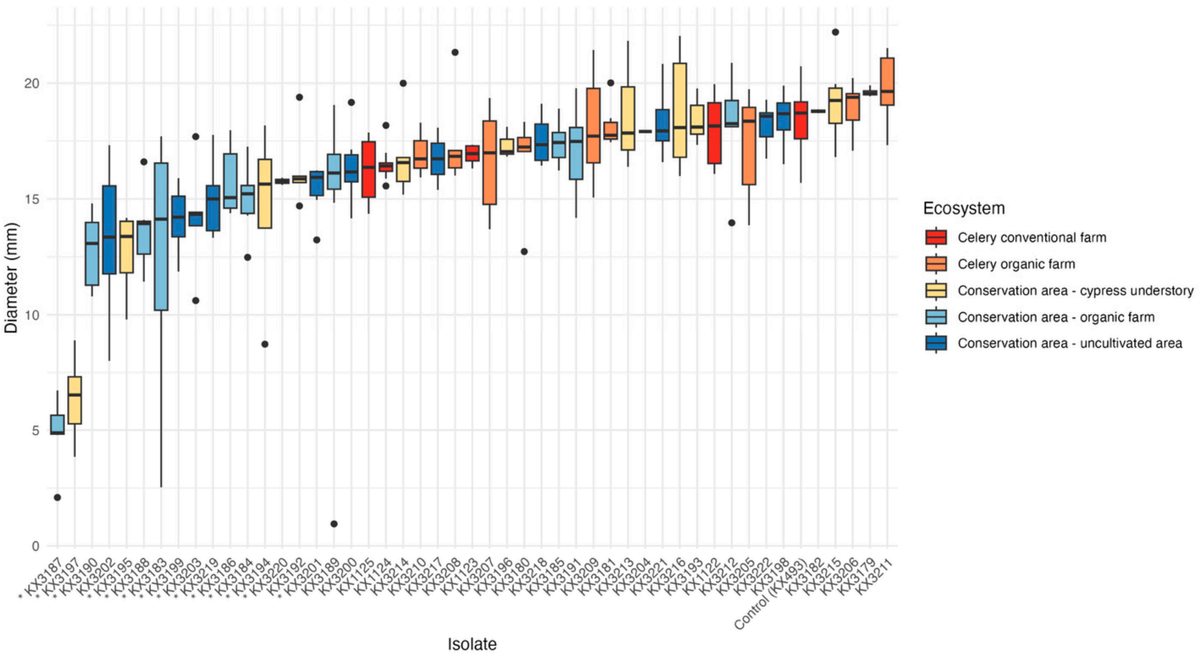

Figure S2. Radial growth (mm) of *Cercospora apii* colonies in dual-culture assays with fungal isolates, measured at 7 days post-inoculation. Isolates marked with an asterisk (*) made direct mycelial contact with *C. apii*. Boxplot colors indicate the ecosystem of origin, and boxes represent the median, interquartile range, and overall distribution of observed values.

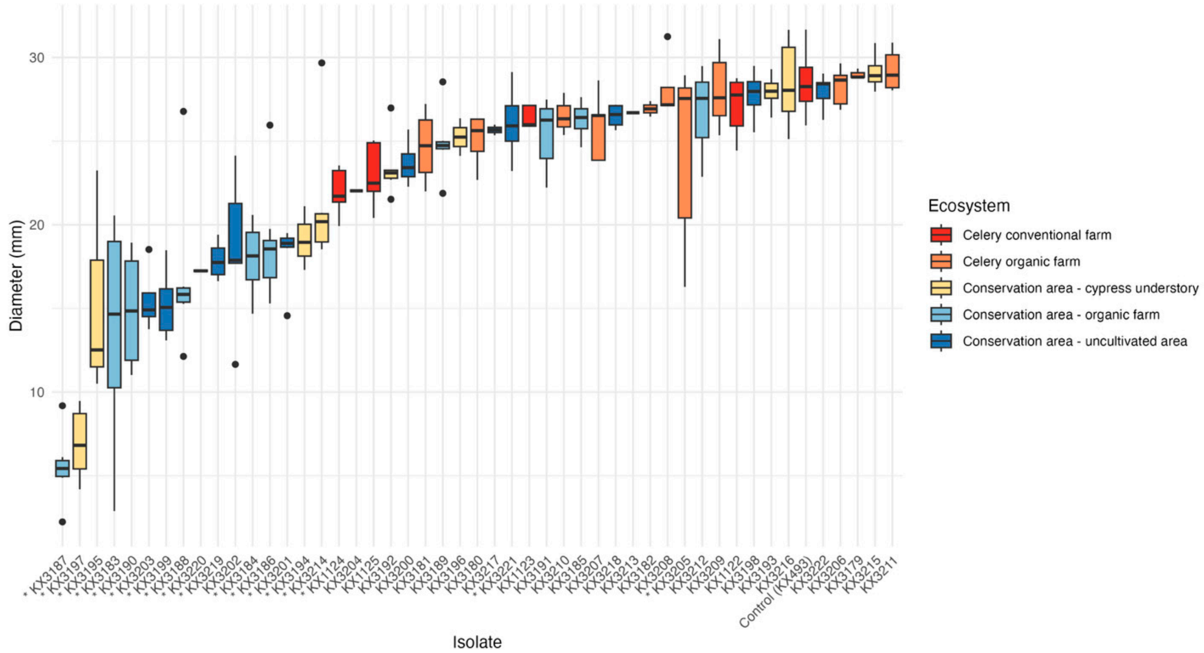

Figure S3. Radial growth (mm) of *Cercospora apii* colonies in dual-culture assays with fungal isolates, measured at 10 days post-inoculation. Isolates marked with an asterisk (*) made direct mycelial contact with *C. apii*. Boxplot colors indicate the ecosystem of origin, and boxes represent the median, interquartile range, and overall distribution of observed values.

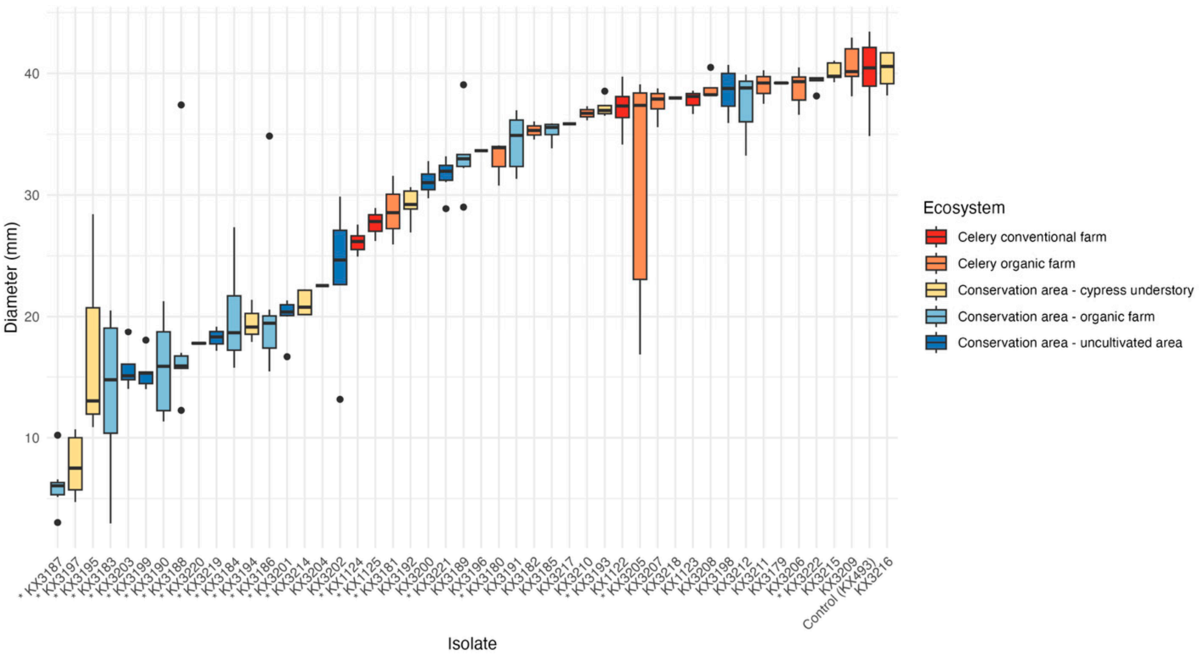

Figure S4. Radial growth (mm) of *Cercospora apii* colonies in dual-culture assays with fungal isolates, measured at 14 days post-inoculation. Isolates marked with an asterisk (*) made direct mycelial contact with *C. apii*. Boxplot colors indicate the ecosystem of origin, and boxes represent the median, interquartile range, and overall distribution of observed values.

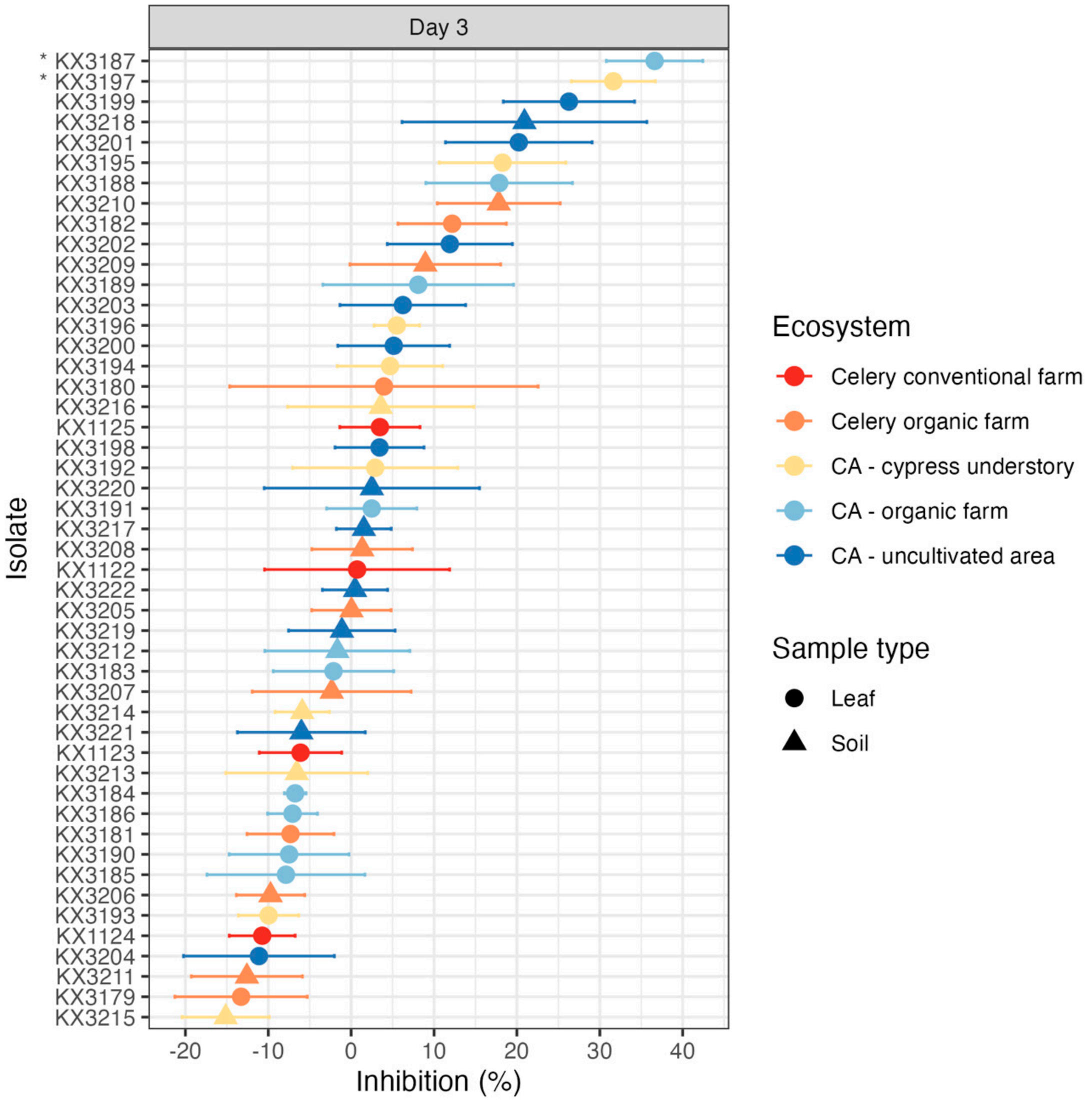

Figure S5. Percentage of *Cercospora apii* mycelial growth inhibition by fungal isolates in dual-culture assays, measured at 3 days post-inoculation. Isolates marked with an asterisk (*) made direct mycelial contact with *C. apii*. Point color indicates the ecosystem of origin, with “CA” referring to conservation areas. Bars represent standard errors of the mean inhibition values.

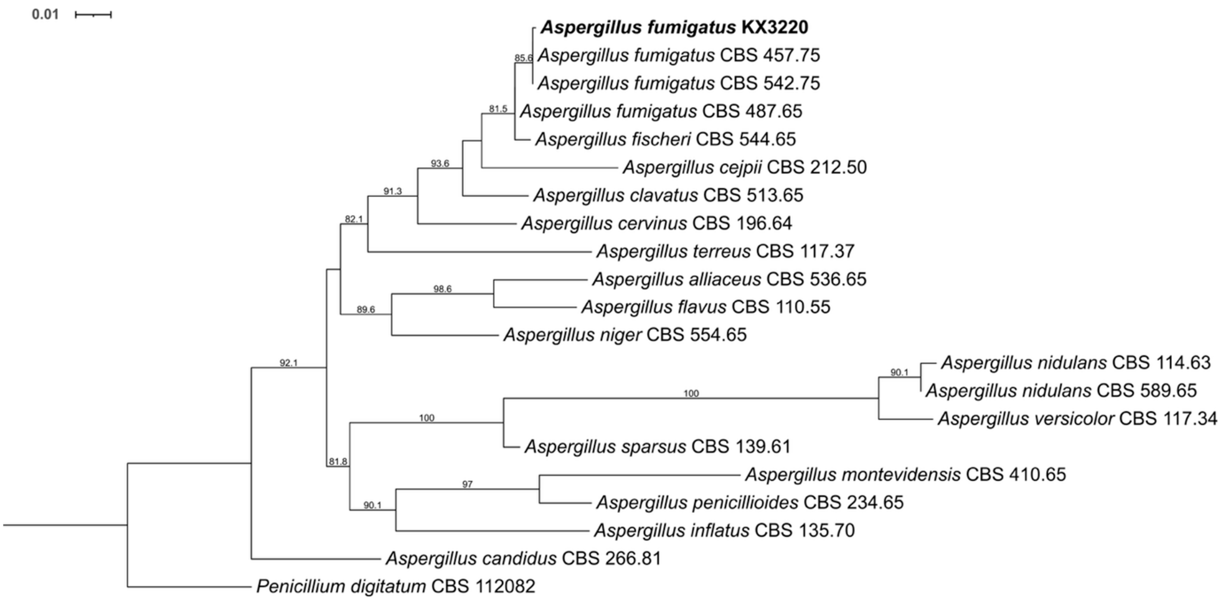

Figure S6. Maximum likelihood phylogenetic tree based on concatenated ITS and LSU sequences, depicting the taxonomic placement of isolate KX3220 as *Aspergillus fumigatus* (highlighted in bold). Bootstrap support values >70% are shown above the branches. *Penicillium digitatum* CBS 112082 was used as the outgroup.

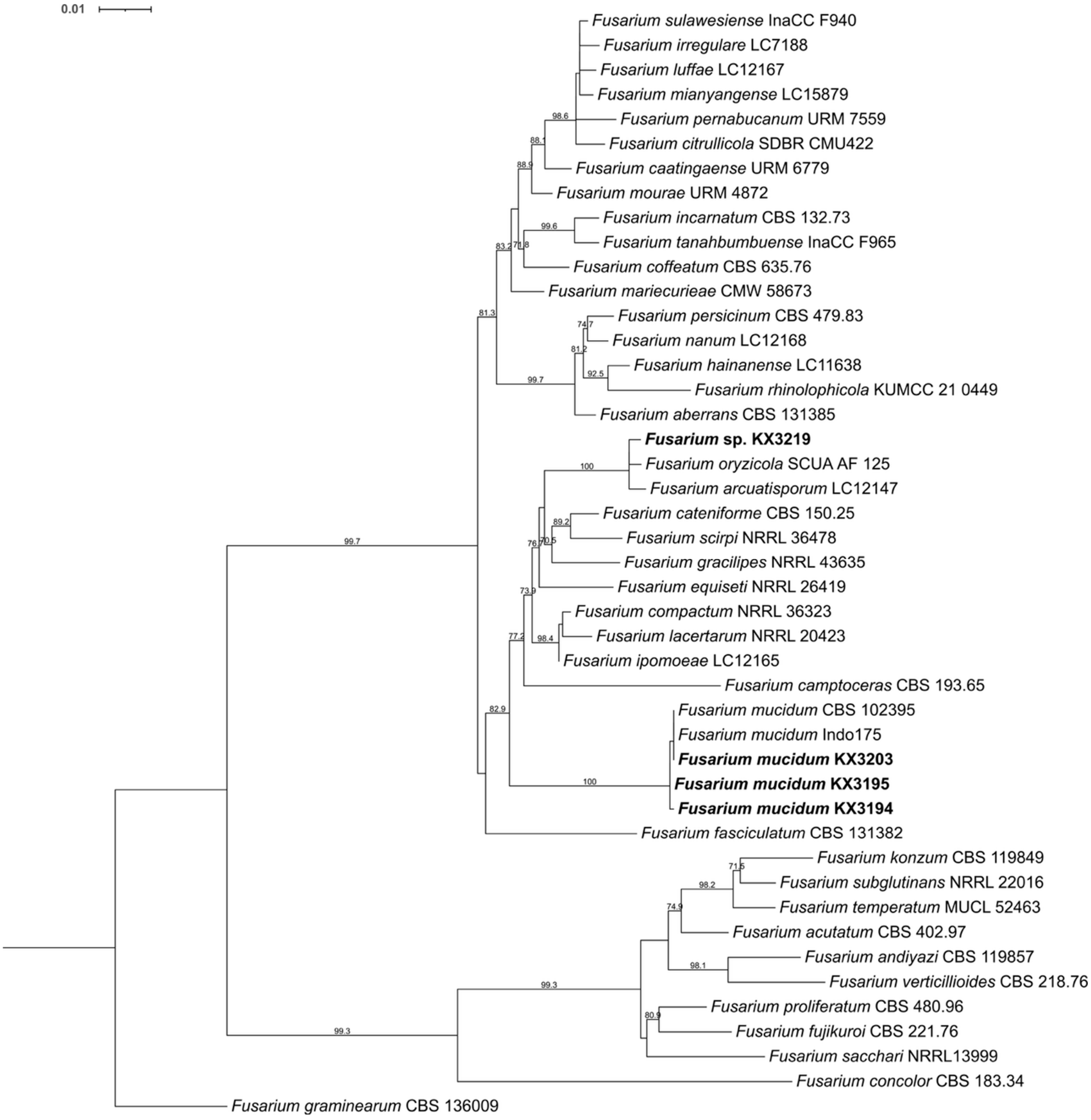

Figure S7. Maximum likelihood phylogenetic tree based on concatenated TEF1 and LSU sequences, depicting the taxonomic placement of *Fusarium* isolates (indicated in bold). Bootstrap support values >70% are shown above the branches. *Fusarium graminearum* CBS 136009 was used as the outgroup.

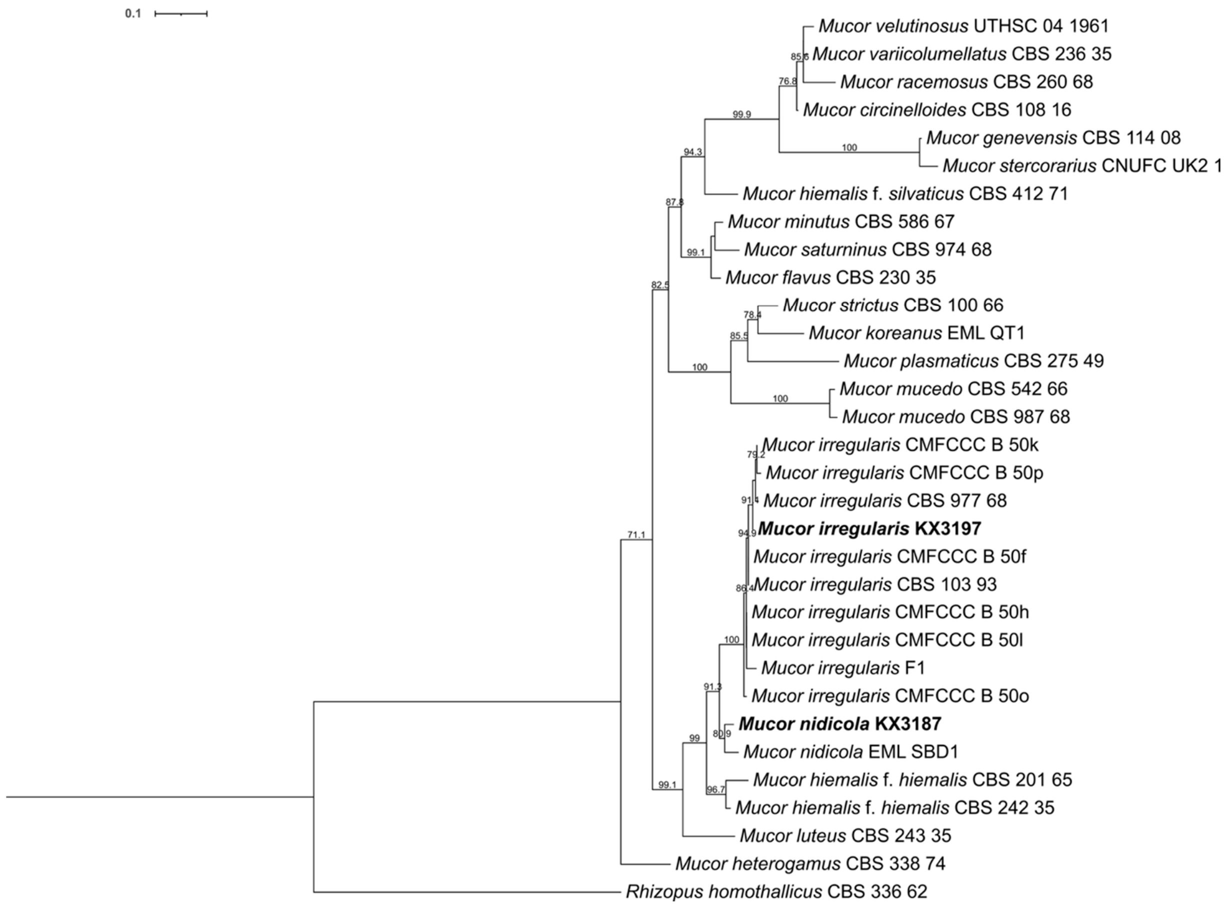

Figure S8. Maximum likelihood phylogenetic tree based on concatenated ITS and LSU sequences, depicting the taxonomic placement of *Mucor* isolates (indicated in bold). Bootstrap support values >70% are shown above the branches. *Rhizopus homothallicus* CBS 336.62 was used as the outgroup.

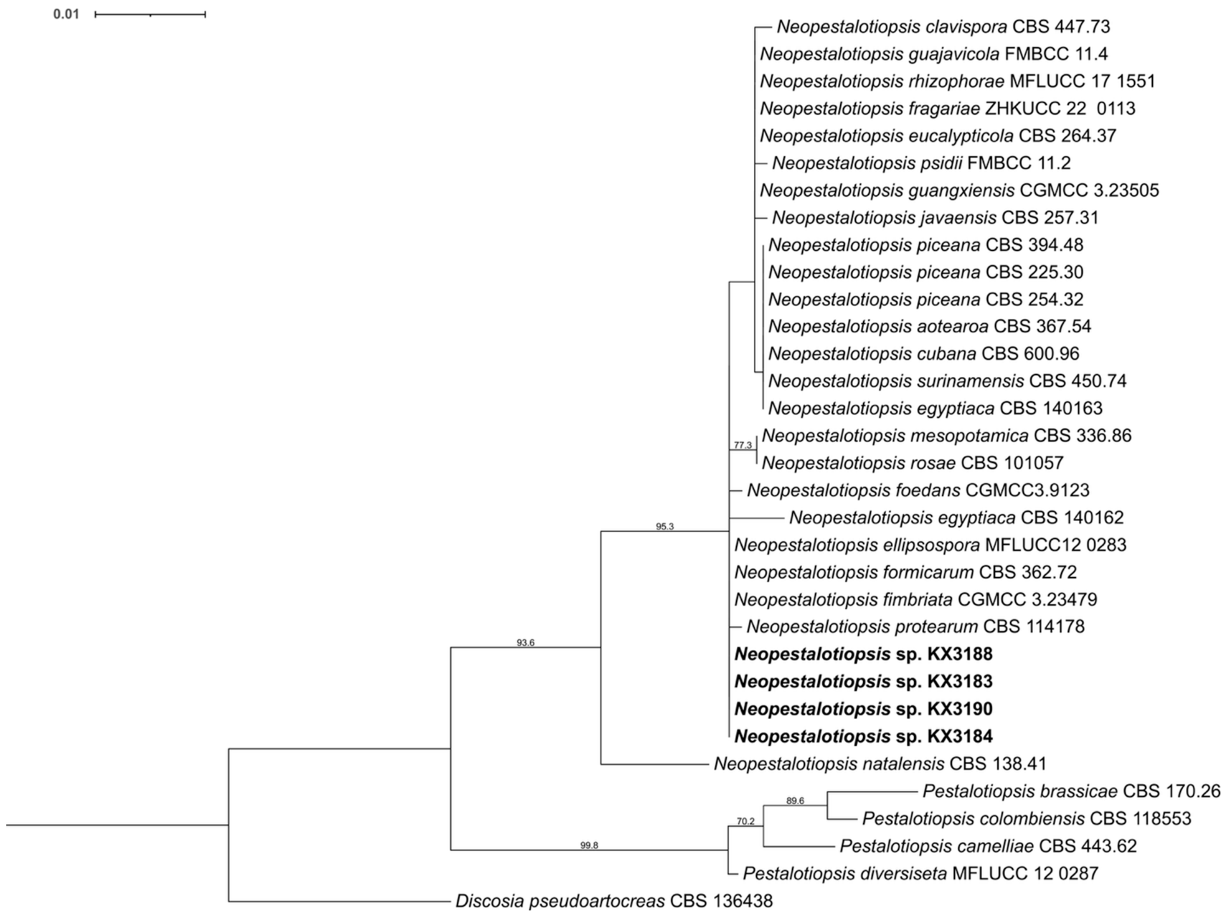

Figure S9. Maximum likelihood phylogenetic tree based on concatenated ITS and LSU sequences, showing the taxonomic placement of *Neopestalotiopsis* isolates (indicated in bold). Bootstrap support values >70% are shown above the branches. *Discosia pseudoartocreas* CBS 136438 was used as the outgroup.

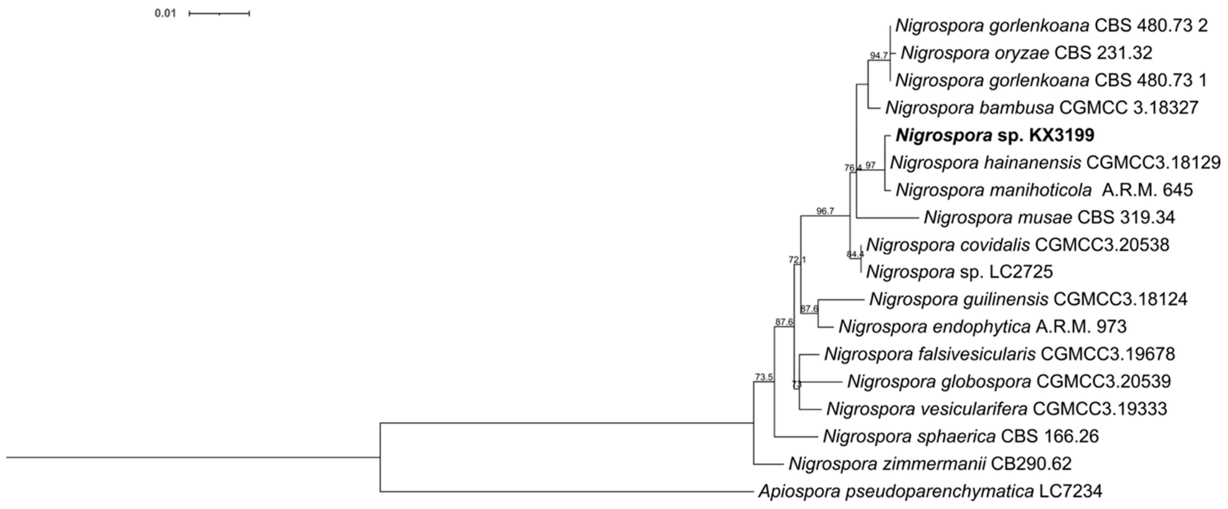

Figure S10. Maximum likelihood phylogenetic tree based on concatenated ITS and LSU sequences, showing the taxonomic placement of *Nigrospora* isolates (indicated in bold). Bootstrap support values >70% are shown above the branches. *Apiospora pseudoparenchymatica* LC7234 was used as the outgroup.

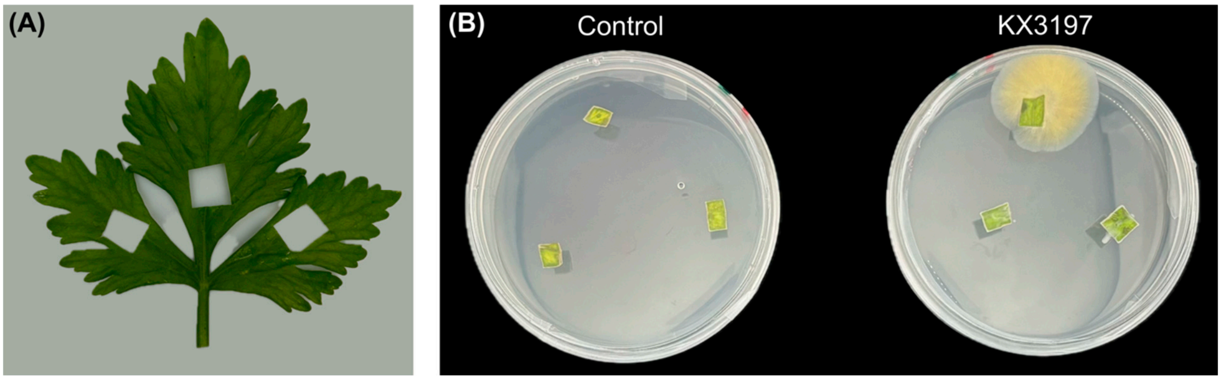

Figure S11. Endophytic colonization assay of celery plants. (A) Representative celery leaf showing the sampling procedure: a ~1 cm^2 segment was excised from each leaflet and surface sterilized prior to plating for endophytic isolation. (B) Representative PDA plates showing no fungal growth in the control (left) and emergence of a characteristic Mucor colony from one leaf segment (right).
